# Pseudologia fantastica: a systematic review of sociodemographic features and psychiatric comorbidities

**DOI:** 10.1097/MS9.0000000000005151

**Published:** 2026-05-15

**Authors:** Tejasvi Kainth, Vasudha Sharma, Ravleen Kaur Suri, Sakshi Prasad, Gurtej Gill, Mona Salehi, Akinahom Asressahegn, Khai Tran, Sasidhar Gunturu

**Affiliations:** a Department of Psychiatry, BronxCare Health System, The Bronx, NY, USA; b Department of Psychiatry, Icahn School of Medicine at Mount Sinai, NY, USA; c Department of Medicine, Dayanand Medical College and Hospital Ludhiana, India; d Department of Forensic Psychiatry, Case Western Reserve University/University Hospitals, Cleveland, OH, USA; e Department of Psychiatry, University of Minnesota, Minneapolis, MN, USA; f Department of Medicine, Saint Paul’s Hospital Millennium Medical College, Addis Ababa, Ethiopia; g Department of Psychiatry, Hackensack Meridian Health, Hackensack, NJ, USA

**Keywords:** demographics, diagnostic difficulty, pseudologia fantastica, psychiatric comorbidity, systematic review

## Abstract

**Background:**

Pseudologia fantastica (PF), or pathological lying, involves elaborate fabrications without clear external gain. Despite its clinical significance, PF lacks recognition in the DSM-5, posing diagnostic and legal challenges.

**Materials and methods:**

A systematic search of PubMed, PsycINFO, and Scopus identified studies on PF demographics, motives, comorbidities, and outcomes. After applying inclusion and exclusion criteria, 34 articles comprising 55 cases were analyzed.

**Results:**

PF predominantly affected men (72.7%), presenting in the third to fourth decades. Major motives included self-aggrandizement (38.2%), attention-seeking (36.4%), and sick-role assumption (29.1%). Common themes were health-related lies (67.3%) and occupational or educational fabrications (43.6%). Cluster B personality disorders (25.5%), depressive disorders (16.4%), and trauma-related disorders (16.4%) were frequent comorbidities. Suicidality (27.3%) and substance use (30.9%) were notable.

**Conclusion:**

PF significantly impacts social, occupational, and legal functioning. Early recognition and clinician–legal awareness are essential for appropriate management.

## Introduction

Pseudologia fantastica (PF), Latin for “fantastic false speech,” is a psychiatric phenomenon characterized by highly elaborate and exaggerated fantasy-like fabrications, with a connection to the individual's own reality as opposed to delusions, made by a person for no apparent reason or personal gain. In the literature, it is also called mythomania, pathological lying, or deception syndrome. These fabrications can encompass aspects such as their identity, academic or occupational background, health conditions, familial or romantic relationships, and events in their life[1]. A “lie” comprises awareness of, intention toward, and a preconceived purpose for a false statement[2]. In contrast, PF involves “believable” falsification based on existing evidence, though disproportionate and exaggerated, and stable over a long period (often years) without a predetermined external goal or profit[3]. PF has an element of dyscontrol while lying; however, the person can acknowledge the falsity of the statements when confronted with facts or truth, as opposed to the fixed belief systems characteristic of delusion^[^1,4^]^.

HIGHLIGHTS

- This systematic review and qualitative analysis explores the demographics of pseudologia fantastica, a rare entity.
- We analyze the psychological factors, namely, the intention behind the fabrications, the content of the fabrications, and the reaction upon confrontation in cases of pseudologia fantastica.
- We determine the psychiatric comorbidities and concurrent substance abuse associated with pseudologia fantastica.

Coined in the late 1800s by Anton Delbrück, a German psychiatrist, PF has not been recognized as an independent psychiatric diagnosis in the Diagnostic and Statistical Manual of Mental Disorders, Fifth Edition (DSM-5)[5]. Theorized to stem from low self-esteem, this syndrome has been mentioned as a facet of post-traumatic stress disorder, factitious disorder, and cluster B personality disorders–narcissistic, antisocial, and histrionic types[6]. The average age of onset for PF is approximately 16 years. Current research on PF presents conflicting findings regarding gender prevalence; some studies suggest that it occurs equally in both genders, while others indicate a higher prevalence in men. Factors such as sociocultural norms, societal expectations surrounding honesty, and regional differences contribute to these gender disparities. Determining the prevalence of PF is particularly difficult because it often appears comorbid with other personality disorders and because individuals with PF are less likely to seek treatment[7].

The intentional fabrications, unconscious motivation, and unclear gain make PF resemble factitious disorder (ICD-10 code: F68.10) the most, which is classified under the DSM-5 category of “Somatic Symptom and Related Disorders”^[^4,5,8^]^. To the extent that 61% of factitious disorders showed evidence of PF and eventual contrived illness[9], the absence of clear profit and the unconscious nature of the motive behind lying differentiates this syndrome from malingering or simple intentional lying. At the same time, the reproducibility and durability of the fabrications over a long time make PF separate from confabulations[10].

The complexity of PF poses a significant diagnostic challenge in filtering the truth from the fantastical fabrications in a clinical setting. Additionally, determining competency to stand trial for a pseudolog poses substantial legal implications, considering that they may inadvertently falsify under oath, which may be caused by the underlying pathological state of mind versus a choice. The complexity of PF presents significant diagnostic and prognostic challenges, particularly in distinguishing truth from the elaborate fabrications characteristic of the condition within clinical settings. These challenges highlight the critical need for an in-depth systematic review of this understudied yet forensically and clinically significant psychiatric phenomenon. A comprehensive understanding of PF and its comorbid neuropsychiatric conditions can facilitate accurate diagnosis and enable tailored, multidisciplinary interventions for better outcomes for patients.

## Methods

### Source of data

**Figure 1.**
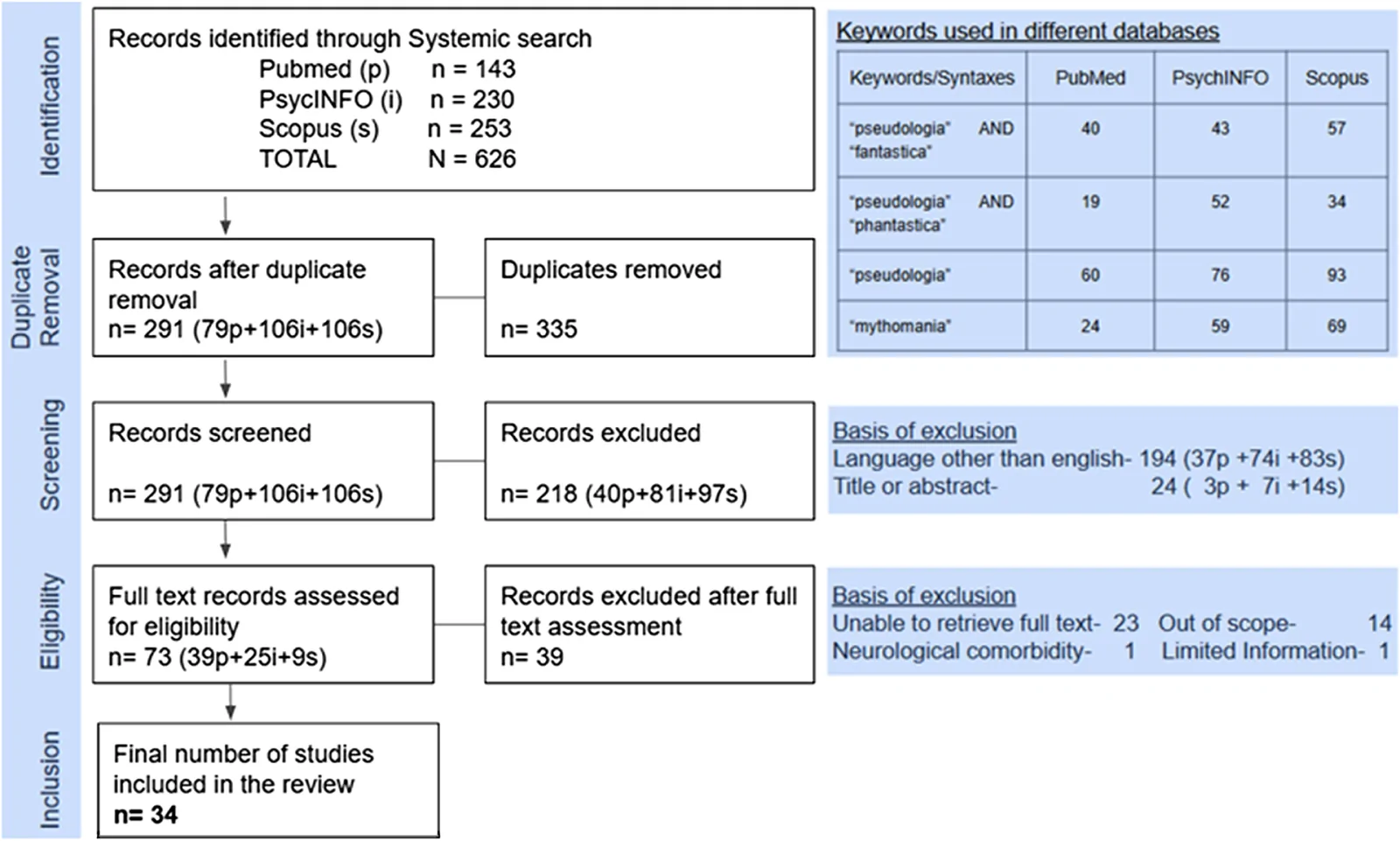
PRISMA flow diagram.

We conducted a systematic search through three databases – PubMed, PsycINFO, and Scopus – following the PRISMA (Preferred Reporting Items for Systematic Reviews and Meta-Analyses) 2020 guidelines. We used the keywords “pseudologia” AND “fantastic,” “pseudologia” AND “phantastica,” “pseudologia,” and “mythomania” and retrieved articles; the details are presented in Figure 1 below.

### Inclusion/exclusion criteria

**Figure 2.**
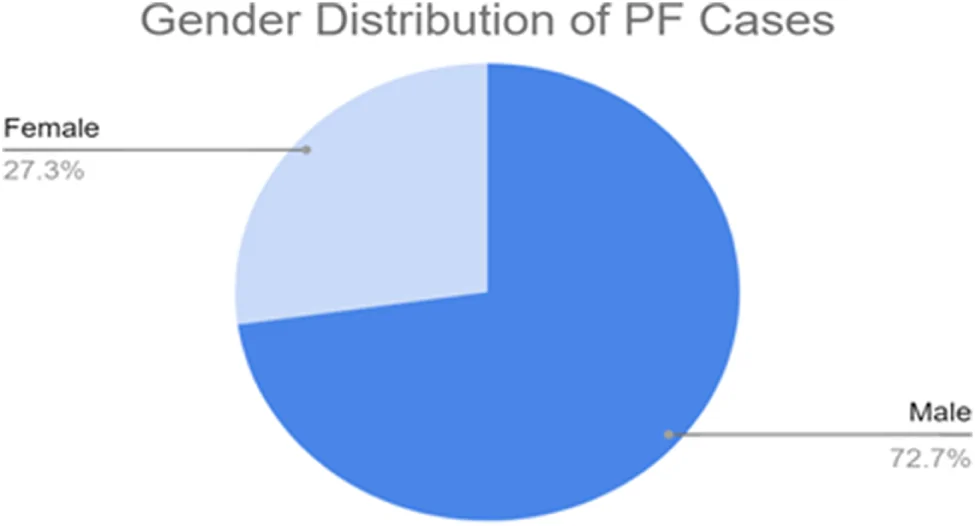
Gender distribution.

We included all original studies published in English, comprising human subjects, from the databases mentioned above, dated between 1914 and 25 January 2024. Our exclusion criteria included all non-English studies, studies with limited information about the cases, studies with neurological comorbidities, and any non-original studies such as letters to the editor, editorials, commentaries, short reviews, narrative reviews, systematic reviews, meta-analytical reviews, or any other review articles. Two researchers independently completed data collection and screening. The collected variables included demographics, substance use, psychiatric comorbidities, and dynamics of the lies told by the patients with PF, as shown in Table 1 and Figure 2. Any dispute regarding inclusion and exclusion criteria for any record analyzed for this systematic research was resolved after an in-depth discussion among the authors and was then independently verified by the third author.

**Table 1:** Patient demographics.

Demographic variable	Estimate (*n*)	Estimate (%; *n*/55 × 100)
Age		
Age range	14–68 years	
Mean age ± SD	35.04 ± 12.71 years	
Age groups (in years)		
10–20^[^11–15^]^	6	10.91%
20–30^[^11,14,16–23^]^	12	21.82%
30–40^[^3,4,10,11,16,24–31^]^	15	27.27%
40–50^[^20,25,29,32–36^]^	10	18.18%
50–60^[^16,29,37–39^]^	5	9.09%
60–70^[^25,33^]^	2	3.64%
Not specified/mentioned	5	9.09%
Gender		
Male^[^3,4,10–14,16–21,24–29,32–35,37,38,40,41^]^	40	72.73%
Female^[^11,15,16,20,22,23,27,29–31,36,39^]^	15	27.27%
Ethnicity		
Caucasian^[^3,10,11,15,20,24,25,28,30,31,39^]^	13	23.64%
AA[16]	1	1.82%
Hispanic[16]	1	1.82%
Asian^[^12,17^]^	2	3.64%
Not specified/ mentioned	38	69.09%
Relationship Status		
Single^[^3,12,15,16,20,21,27,30,35^]^	10	18.18%
In a relationship/dating[13]	1	1.82%
Married^[^11,24,25^]^	4	7.27%
Divorced/separated^[^4,10,20,25,26,31,34,37,39^]^	10	18.18%
Widowed[16]	1	1.82%
Not specified/mentioned	29	52.73%
Educational level		
Uneducated/high school dropout^[^3,10,12,21,25,32^]^	7	12.73%
Current student^[^13,15^]^	2	3.64%
High school grad^[^17,20,24,27,35,37^]^	6	10.91%
Professional degree[18]	1	1.82%
GED/trade school^[^4,39^]^	2	3.64%
Not specified/mentioned	37	67.27%

### Data extraction

We retrieved a total of 626 records. After removing the duplicates, we recovered a total of 291 articles. We included all original studies on human participants, including case reports, case series, and retrospective chart reviews. We could not retrieve the full text for 23 records, so those studies were excluded from the assessment. After applying the title, abstract, and full-text evaluation, a total of 34 articles, consisting of 55 cases of PF, were included in our final study (Fig. 1).

### Data analysis

Data were analyzed using STATA MP17 (StataCorp, College Station, TX, USA). Continuous variables were presented as mean ± standard deviation, and categorical variables were presented as frequency (percentage). The Shapiro-Wilk test was used to assess the normality of the data, which showed a normal distribution. All comparisons between the two gender groups for variables were performed using chi-square tests. A *P*-value less than 0.05 was considered statistically significant.

## Results

### Patient demographics

**Figure 3.**
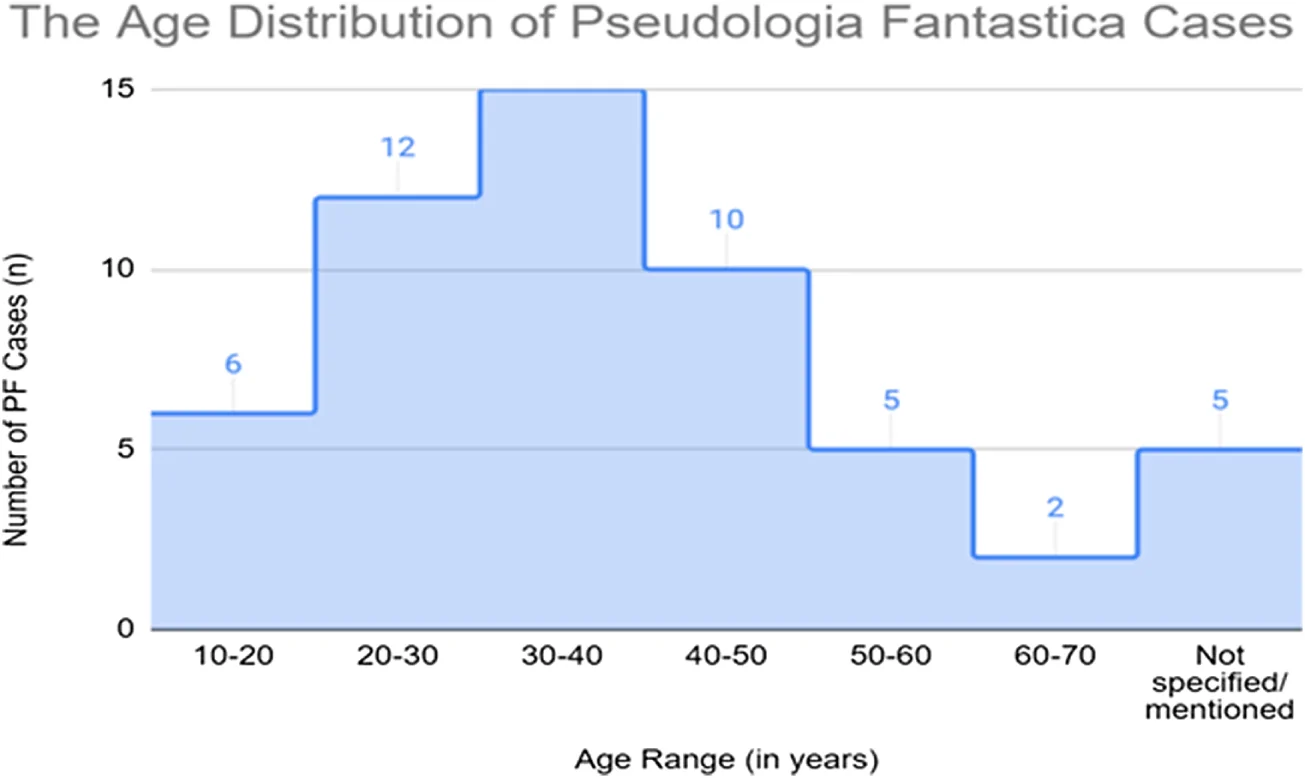
Age distribution.

The 34 final studies we included in the review provided us with a sample consisting of 55 PF cases, with their demographics shown in Table 1. Seventy-three percent of these were men^[^3,4,10–14,16–21,24–29,32–35,37,38,40,41^]^, and 27% were women^[^11,15,16,20,22,23,27,29–31,36,39^]^ (Fig. 2), with the average age being 35.04 ± 12.71 (mean age ± SD) years. The age ranged from 14 to 68 years, with 49.09% of cases presenting to medical institutions between 20 and 40 years of age, as seen in Figure 3[3,4,10,11,14,16–31^]^. The ethnicities in most cases (69.06%) were not specified.

### Content of fabrications

**Figure 4.**
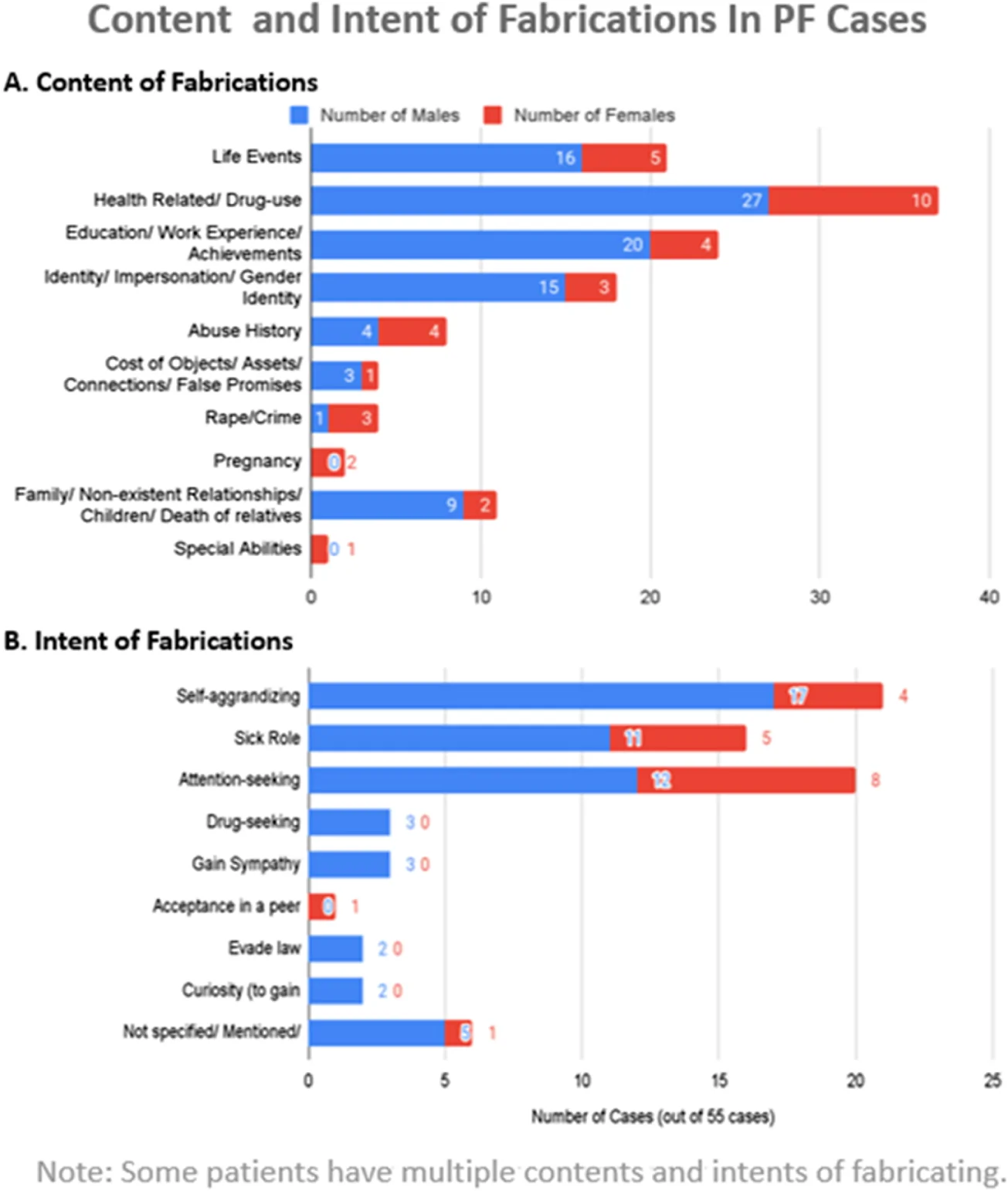
Frequency comparison of content of fabrications in PF (top), frequency comparison of intent of PF lies (bottom).

The documented cases had a variety of fabrications that we enlisted in our database. The fabrications were based on several facets of their lives. We grouped them into different categories depending on their content (Table 2). The fabrications in 67.27% of the PF cases were predominantly health- or drug-use-related ^[^3,4,10,11,14,18–21,23–33,36–39,41^]^, followed by 43.64% of cases that falsified their educational or work-related achievements. Other patterns documented are categorized in Figure 4.

**Table 2:** Content of fabrications in PF cases.

Content	Number of cases (*n*, out of 55 cases)	Percentage of total (%, *n*/55 × 100)	Number of males (*m*, out of 40)	Percentage of males (%, *m*/40 × 100)	Number of females (*f*, out of 15)	Percentage of females (%, *f*/15 × 100)	*P*-value
Life events^[^4,10,11,13–16,19–21,24,25,29,33,34,37,39,40^]^	21	38.18%	16	40%	5	33.33%	0.65
Health related/drug-use^[^3,4,10,11,14,18–21,23–33,36–39,41^]^	37	67.27%	27	67.5%	10	66.67%	0.95
Education/work experience/achievements^[^3,4,12,14,16,17,19–21,24,25,27,28,31,33,36,37,39,40^]^	24	43.64%	20	50%	4	26.67%	0.12
Identity/impersonation/gender identity^[^3,12,14,16–18,20,26,27,29,30,32,33,36,40^]^	18	32.73%	15	37.5%	3	20%	0.21
Abuse history^[^4,11,16,24,26,30,34,39^]^	8	14.54%	4	10%	4	26.67%	0.11
Cost of objects/assets/connections/false promises^[^11,14,16^]^	4	7.27%	3	7.5%	1	6.67%	0.91
Rape/crime^[^11,16,22^]^	4	7.27%	1	2.5%	3	20%	0.02
Pregnancy^[^11,31^]^	2	3.64%	0	0%	2	13.33%	0.01
Family/non-existent Relationships/children/death of relatives^[^12,16,17,19,23,25–27,29^]^	11	20.00%	9	22.5%	2	13.33%	0.45
Special abilities[15]	1	1.82%	0	0%	1	6.67%	0.09

Some patients have multiple contents of fabrications.

In Table 2, we also contrasted the contents of the fabrications between men and women. We found that patterns related to rape/crime (*P* = 0.02) and pregnancy (*P* = 0.01) were observed more frequently in women compared to men. Abuse history was falsified more often by women than men (26.7% vs. 10%). Men, on the other hand, exaggerated more about education/work experience/achievements (50%) and their identity/impersonation/gender identity (37.5%) than women (26.67% and 20%, respectively). However, these comparisons were not statistically significant.

### Intent of fabrications

The 55 reviewed cases had multiple intentions behind fabrications, often co-occurring. We classified their motives into nine different categories (Table 3) and, in some cases, interpreted the intention behind the fabrication by qualitatively analyzing the cases that did not mention the intent explicitly. In Table 3, we also compared the intentions distinctly for men and women. We found that women are more likely to seek attention (53.33%) than men (30%). Men exhibited more self-aggrandizing traits (42.5%) than women (26.67%). However, these comparisons were not statistically significant after analysis.

**Table 3:** Intent of falsification in PF cases.

Intent	Number of cases (*n*, out of 55 cases)	Percentage of total (%, *n*/55 × 100)	Number of males (*m*, out of 40)	Percentage of males (%, *m*/40 × 100)	Number of females (*f*, out of 15)	Percentage of females (%, *f*/15 × 100)	*P*-value
Self-aggrandizing^[^3,4,10,11,13–16,20,21,24,25,33,35–37^]^	21	38.18%	17	42.5%	4	26.67%	0.28
Sick role^[^11,16,20,25,28,29,31,33,36,38,41^]^	16	29.09%	11	27.5%	5	33.33%	0.67
Attention-seeking^[^10–12,14–16,19–23,26,27,31,32,39^]^	20	36.36%	12	30%	8	53.33%	0.10
Drug-seeking^[^17,28,33^]^	3	5.45%	3	7.5%	0	0%	0.27
Gain sympathy^[^10,21,29^]^	3	5.45%	3	7.5%	0	0%	0.27
Acceptance in a peer group[15]	1	1.82%	0	0%	1	6.67%	0.09
Evade law^[^25,40^]^	2	3.64%	2	5%	0	0%	0.37
Curiosity (to gain experience)/gain pleasure^[^14,40^]^	2	3.64%	2	5%	0	0%	0.37
Not specified/mentioned/unclear^[^18,25,27,30,33,34^]^	6	10.91%	5	1.25%	1	6.67%	0.53

Some patients have multiple intents of lying.

In some cases, the intent of the lie was interpreted by co-authors via qualitative analysis of the cases.

### Reaction to the first confrontation

The documented cases had a diverse range of reactions recorded during their first confrontation about their lying patterns (Table 4). Most cases (36.36%) admitted or confessed to their falsification tendencies^[^10–15,19–23,27,29,30,33,37,38,40^]^, followed by 15 cases (27.27%) modifying their fabrications or deflecting to an alternative collateral.

**Table 4:** Reaction of patients with PF on confrontation.

Reaction of first confrontation	Number of cases (*n*, out of 55 cases)	Percentage of total (%, *n*/55 × 100)	Number of males (*m*, out of 40)	Percentage of males (%, *m*/40 × 100)	Number of females (*f*, out of 15)	Percentage of females (%, *f*/15 × 100)	*P*-value
Modifying the lie/deflected to an alternate collateral^[^4,11,13,14,17,19–21,24–26,32,34,35,40^]^	15	27.27%	15	37.5%	0	0%	0.005
Retreating from the lie/evasive/escape the situation/passed the blame^[^4,10,11,25,29,41^]^	8	14.55%	6	15%	2	13.33%	0.87
Anger/argumentative/acted Out^[^10–12,28–30^]^	13	23.64%	8	20%	5	33.33%	0.30
Depressed/withdrawn/regretful^[^11,20^]^	2	3.64%	1	2.5%	1	6.67%	0.46
Admitted to lying/accepted/reluctant agreement/self-confession^[^10–15,19–23,27,29,30,33,37,38,40^]^	20	36.36%	15	37.5%	5	33.33%	0.77
Faltered/confusion^[^11,28,30,32,37^]^	5	9.09%	3	7.5%	2	13.33%	0.50
Inconsistent lie/contradictory lies^[^14,16,25,28,30^]^	5	9.09%	4	10%	1	6.67%	0.70
Continued lying^[^10,12,15,30^]^	4	7.27%	2	5%	2	13.33%	0.28
Deny that it is a lie^[^14,20,25,26,28,31–33^]^	7	12.73%	5	12.5%	2	13.33%	0.93
Dissociation[11]	1	1.82%	0	0%	1	6.67%	0.09
Not confronted/reaction not mentioned^[^3,12,16,18,26,29,33,36,39^]^	11	20.00%	7	17.5%	4	26.67%	0.44

Some patients have multiple reactions to confrontation.

In Table 4, we juxtaposed the reactions of male and female patients affected by PF when confronted about their fabrications. We found that men modify the initial falsification or deflect to alternate collateral upon confrontation (37.5%) more than women (*P* < 0.01). We also found that more women (33.33%) expressed anger, argued, or acted out than their male counterparts (20%), although this difference was statistically insignificant.

### Psychiatric comorbidities

**Figure 5.**
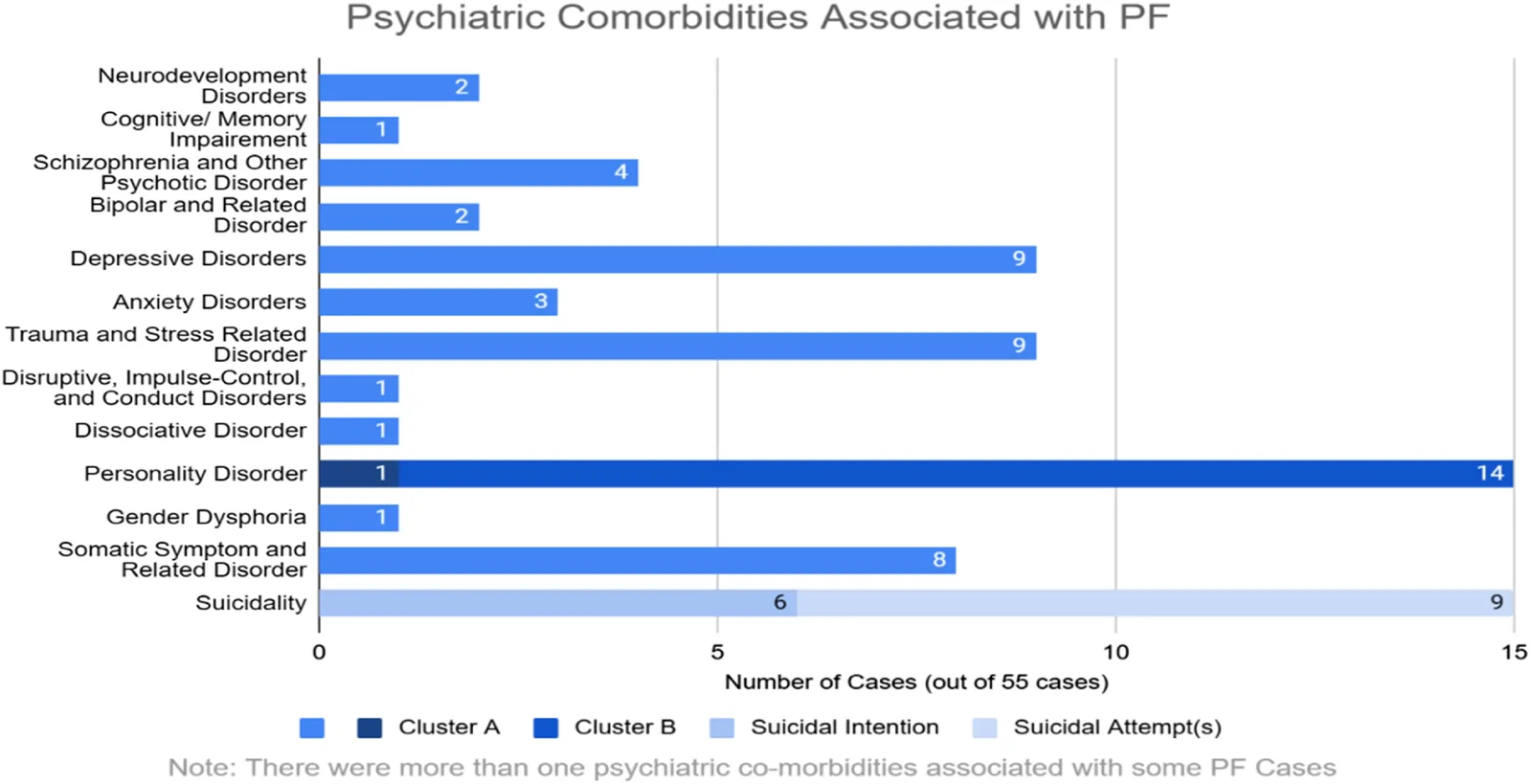
Psychiatric comorbidities.

As shown in Table 5, several psychiatric comorbidities were also reported in the included documentation of patients with PF. These comorbidities were divided according to the standard classification system of mental disorders in the DSM-5. Fifteen of the total 55 PF cases had a concurrent personality disorder (27.27%)^[^11,16,21,27,29,30,32,33,37^]^, the majority of them (14 out of 15 cases) exhibiting Cluster B personality traits^[^11,16,21,27,29,30,32,33,37^]^, and only 1 out of 15 being classified as Cluster A personality[37]. We also found single cases reported with disruptive, impulse control, and conduct disorders[13], dissociative disorder[11], and gender dysphoria[12]. Besides these psychiatric comorbidities, one case also presented with cognitive or memory impairment[25] (Figure 5).

**Table 5:** Psychiatric comorbidities in PF cases.

Effect on QoL	Number of cases (*n*, out of 55 cases)	Percentage of total (%; *n*/55 × 100)
Neurodevelopmental disorders^[^13,15^]^	2	3.64%
Cognitive/memory impairment[25]	1	1.82%
Schizophrenia and other psychotic disorders^[^10,16,34,38^]^	4	7.27%
Bipolar and related disorders^[^16,22^]^	2	3.64%
Depressive disorders^[^11,16,17,24,25,27,30^]^	9	16.36%
Anxiety disorders^[^24,27,37^]^	3	5.45%
Trauma and stress related disorders^[^4,13,22,24,32,34,37–39^]^	9	16.36%
Disruptive, impulse-control, and conduct disorders[13]	1	1.82%
Dissociative disorders[11]	1	1.82%
Personality disorders	14	25.45%
Cluster A[37]	1	1.82%
Cluster B^[^11,16,21,27,29,30,32,33,37^]^	14	25.45%
Gender dysphoria[12]	1	1.82%
Somatic symptom and related disorders^[^3,11,26,28,29,37^]^	8	14.54%
Suicidality	13	23.64%
Suicidal intention^[^13,16,27,32,33,39^]^	6	10.91%
Suicidal attempt(s)^[^11,13,16,21,30,34,35,37^]^	9	16.36%

There were more than one psychiatric comorbidities associated with some PF cases.

### Substance use

**Figure 6.**
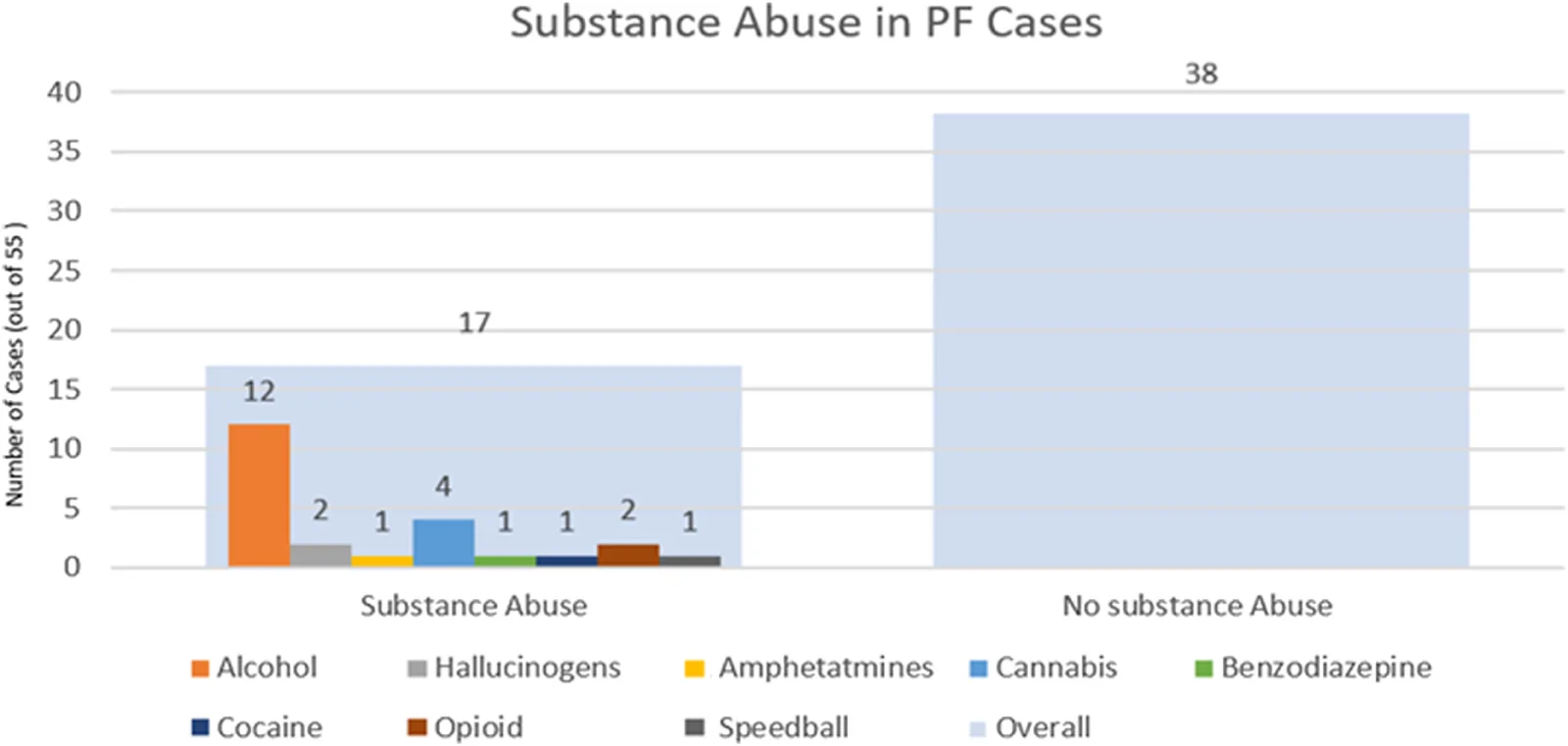
Substance abuse.

Most reviewed cases (69.09%) did not mention a substance/drug abuse problem, as seen in Table 6 and Figure 6. However, in the instances that described patients being exposed to substance or drug use, the majority of them addressed alcohol abuse, which accounted for 21.82% of the total cases^[^4,10,11,14,24,25,30,33,36,37^]^.

**Table 6:** Substance use associated with PF cases.

Effect on QoL	Number of cases (*n*, out of 55 cases)	Percentage of total (%; *n*/55 × 100)
Substance abuse	17	30.91%
Alcohol^[^4,10,11,14,24,25,30,33,36,37^]^	12	21.82%
Hallucinogens^[^4,11^]^	2	3.64%
Amphetamines[4]	1	1.82%
Cannabis^[^4,11,24,37^]^	4	7.27%
BZD[11]	1	1.82%
Cocaine[11]	1	1.82%
Opioids^[^17,32^]^	2	3.64%
Speedball[11]	1	1.82%
No substance use mentioned	38	69.09%

Some patients with PF had more than one substance they abused.

### Effect of PF on quality of life

**Figure 7.**
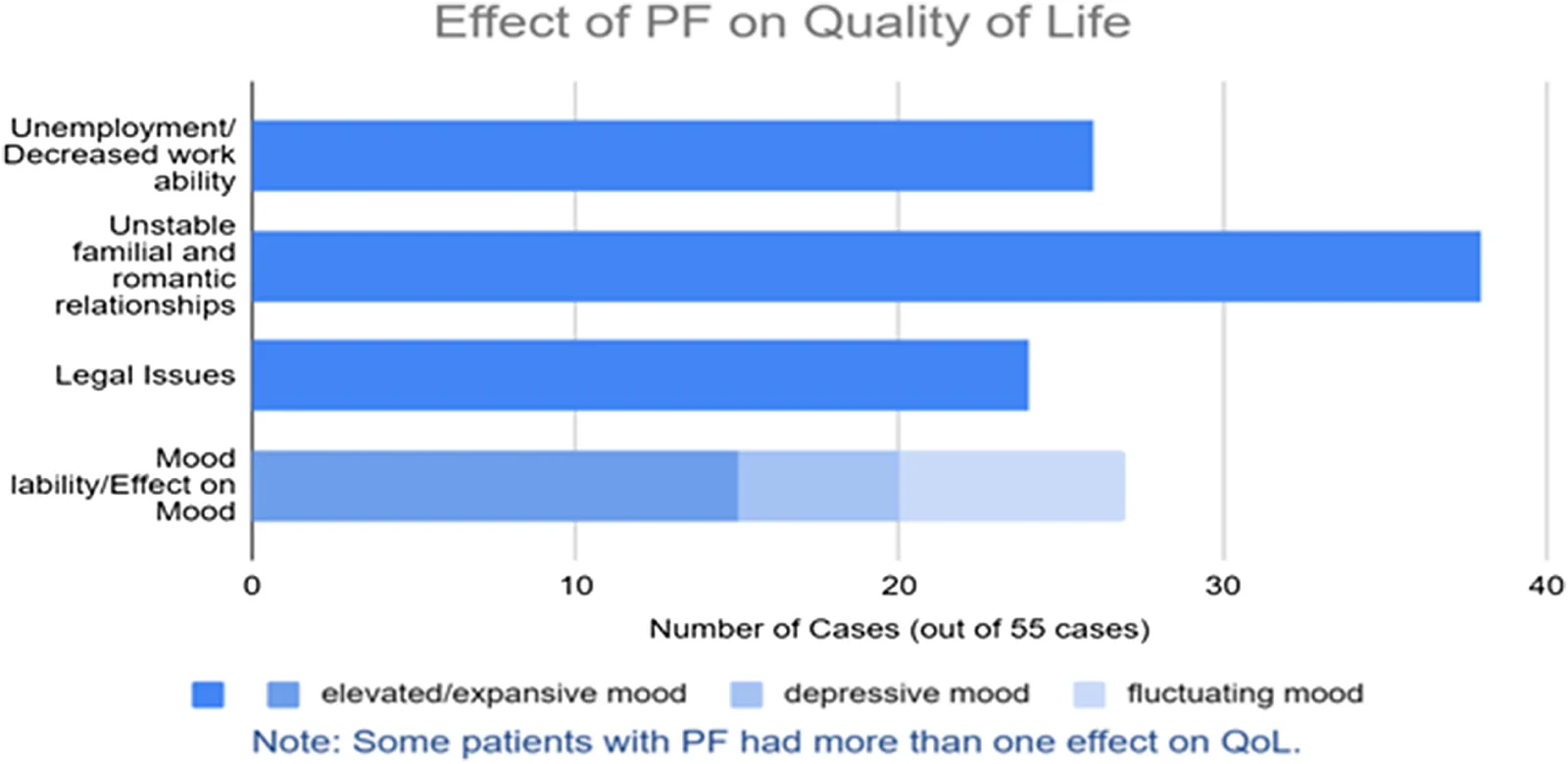
Effect on quality of life (QoL).

PF was found to have a markedly negative impact on quality of life (Table 7). The majority of individuals (69.09%) experienced unstable familial or romantic relationships^[^3,4,10–15,17–21,23–35,37,39,40^]^. Furthermore, 47.27% of the cases involved unemployment or decreased work ability^[^4,10,14,16,17,19–22,25,27,29,30,33,35–37,39,40^]^, and 43.64% faced legal issues or similar implications^[^3,4,11,13,14,16,18,20,22,25,26,29,31–33,35–37,39,40^]^, as illustrated in Figure 7.

**Table 7:** Effect of PF on quality of life.

Effect on QoL	Number of cases (*n*)	Percentage of total (%, *n*/55 × 100)	Number of males (*m*, out of 40)	Percentage of males (%, *m*/40 × 100)	Number of females (*f*, out of 15)	Percentage of females (%, *f*/15 × 100)	*P*-value
Unemployment/decreased work ability^[^4,10,14,16,17,19–22,25,27,29,30,33,35–37,39,40^]^	26 out of 55 cases	47.27%	20	50%	6	40%	0.50
Unstable familial and romantic relationships^[^3,4,10–15,17–21,23–35,37,39,40^]^	38 out of 55 cases	69.09%	28	70%	10	66.67%	0.81
Legal issues/consequences^[^3,4,11,13,14,16,18,20,22,25,26,29,31–33,35–37,39,40^]^	24 out of 55 cases	43.64%	18	45%	6	40%	0.73
Mood lability/effect on Mood^[^4,10–15,17,18,21,24,26,29,30,32–34^]^	27 out of 55 cases	49.09%	20	50%	7	46.67%	0.82
Elevated/expansive mood^[^11,12,14,15,17,18,21,26,29,33^]^	15 out of 27 cases	55.55%	11	27.5%	4	26.67%	0.95
Depressive mood^[^4,11,26,30,32^]^	5 out of 27 cases	18.52%	3	7.5%	2	13.33%	0.50
Fluctuating mood^[^10,11,13,14,24,33,34^]^	7 out of 27 cases	25.93%	6	15%	1	6.67%	0.40

Some patients with PF had more than one effect on QoL.

In Figure 7 and Table 7, we examined the differences between men and women regarding the effect of PF on quality of life. We found statistically insignificant minor variations between the two genders.

## Discussion

Despite the inherent difficulty in its clinical management and legal ramifications, PF has scarce associated data and research, with some considering it a “pretentious synonym” for abnormal lying[42]. Combining the three databases mentioned in the methodology section, we found 55 unique cases of PF in the existing literature. In our study, approximately 49% of the PF cases presented within the age range of 20–40 years, with the mean age being 35.04 ± 12.71 years. A Scandinavian study reported the mean age of patients with PF to be 22 years, which is one standard deviation lower than our reported mean age range[1]. It has also been reported that pseudologia first manifests in adolescence^[^1,43^]^, which corresponds to our findings, with a prevalence of 10.9% in the age group of 10–20 years. We found a male predominance (72.73%) in our report, and studies have reported parallel observations in the gender distribution related to the frequency of dishonesty^[^33,44–46^]^. However, reports have suggested contrasting data, where equal male-to-female distribution^[^1,47^]^ and a female skew^[^43,48^]^ were observed, suggesting that social and cultural expectations for honesty and deception across geographical areas may affect gender variation in those regions [47]. We found no associations between PF and demographics such as ethnicity, educational level, and marital status mentioned in the existing literature.

In light of the close similarity with factitious disorder (4), we observed that health-related embellishments were the most common (67.27%). This was followed by overstated accomplishments (43.64%) and life events (38.18%), which is in line with the criterion of the pseudolog being portrayed as a “hero”^[^1,3,49^]^. Impersonation was present in 32.73% of our PF cases, albeit the third most prevalent content of lies has serious legal consequences^[^3,40^]^. King *et al* observed similar findings in 31% of cases, and 25% of Munchausen syndrome cases were associated with patients changing their names^[^1,33^]^. Clinically, personal achievements were exaggerated more by men, and abuse history was falsified more by women. Men impersonated significantly more. Fabrications about rape/crime (*P*-value = 0.02) and pregnancy (*P*-value = 0.01) were significantly higher in women, the latter of which was expected, as it is possible only in biological women. While these differences have not been studied in the context of PF, in narcissistic disorder, it has been extensively researched that gendered parenting, societal roles, and hormonal exposure (2D:4D ratio) result in “vulnerable women” and “grandiose men.” A similar ego-centric model can also be theorized for the content of PF lies, which needs to be studied further ^[^50,51^]^.

**Figure 8.**
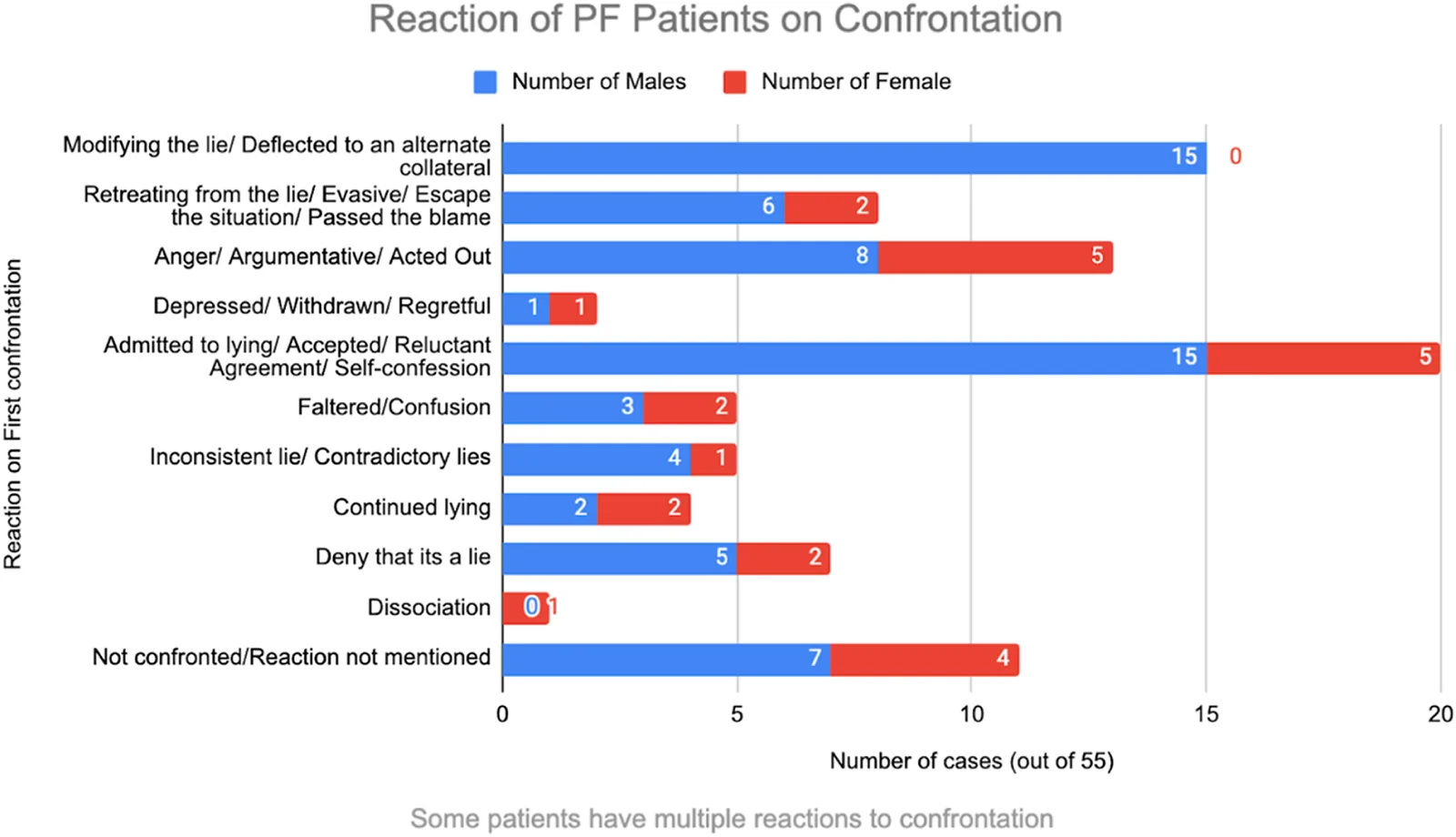
Reaction to first confrontation.

Self-aggrandizing seemed to be the most common (38.18%) intent behind the falsifications in our review, with vanity being the prevailing internal purpose of pseudologia in other studies as well^[^1,9,14^]^. The sick role appeared to be another primary (29.09%) goal of people affected by PF, further strengthening the link to factitious disorders. This was similar to the 25% of pseudologs simulating disease observed in the existing literature[1]. The need for attention emerged as a critical intention among 36.36% of cases, which has yet to be reviewed. We found that self-aggrandizing was more common in men (42.5% in men vs. 26.67% in women) and attention-seeking in women (53.33% in women vs. 30% in men), which is notable clinically despite statistical insignificance. Admission to their frequent falsifications (36.36%) showcases an awareness and insight into their pathology. The ability to modify their fabrications (27.27%), retreating when confronted (14.55%), and inconsistency between the falsifications (9.09%) hint toward the yielding and flexible character of their concepts. As seen in Figure 8, the reactions to confrontation present a contradistinction to the false, fixed, and firm nature of a delusion seen in psychotic disorders in DSM-5. The awareness of their deceit sets PF apart from Somatic Symptom and Related Disorders (excluding factitious disorder)[10]. Modifying fabrications was significantly more common in men (*P*-value = 0.005), whereas clinically, women were more prone to acting out or becoming angry (33.33% in women vs. 20% in men). Personality disorders, especially cluster B (25.45%), were a psychiatric comorbidity present in 27.27% of PF cases, explaining why PF is mentioned as a feature of cluster B personality disorders in the DSM-5^[^5,20^]^. Suicidality was seen to be prevalent (27.27%) in the pseudologs, with 16.36% attempting suicide, similar to the 15% rate of suicide attempts in PF as reported by King *et al*[1]. Suicidal attempts have also been found to be common in Munchausen syndrome[20]. Substance use has been linked with 26%–33.9% of PF cases^[^1,33^]^, as reflected in our review, with 30.91% of cases having any type of substance use, including 21.82% alcohol use.

PF significantly affects the quality of life, resulting in unsteady employment^[^14,20^]^ and legal complications^[^1,3,20,33,40^]^. Trust is fundamental to interpersonal relationships, and PF is often associated with antisocial behavior or social isolation^[^20,33,52,53^]^. It is worth noting that truth is not a cultural necessity but a socially acceptable version of reality vis-à-vis the perception of being truthful, even if it is untrue[54]. We found unemployment (47.27%), unstable interpersonal relationships (69.09%), and legal issues/consequences (43.64%) in our review. The effect of PF on quality of life was comparable among both genders, with minor variations. A slight modification of the criterion for factitious disorder to include falsifications of all types, in addition to bodily symptoms, can result in the easy inclusion of PF in DSM-5[55]. The significant challenges in clinical and legal environments and the superfluous use of medical, social, forensic, and legal resources[36] make it imperative to give PF its due consideration.

## Study limitations

This review had a few limitations that restricted a thorough analysis of PF and slightly hindered the interpretation of the findings. First, we enlisted the documented cases dating back to the early 1900s to encompass as much information as possible about this psychiatric rarity. However, we must recognize that the diagnostic criteria, including the DSM, have been updated numerous times since then, leading to a marked variation in the terminology and concepts used in the studies published over a wide range of time. This lack of a standard diagnostic criterion for PF may have led to inconsistencies in the data.

Restricting our research to only English might have resulted in bias, impeding our research. Moreover, the heterogeneity of the patient demographics, including patient education, ethnicity, and marital status, should have been more extensively mentioned in the existing literature. This limited our ability to conclude on those factors.

The screening of selected databases resulted in a limited number of studies and, therefore, a smaller sample size (55 cases) for our review. This would result in low statistical power and less generalizability of our results. Additionally, due to the small sample size, our attempt at a gender-based comparison for the variables in this study yielded insignificant results, despite the percentages showing a clinical variation between men and women. This limitation highlights the need for more original research on this syndrome.

Last, we had to interpret the intention behind the fabrication by qualitatively analyzing the cases that did not mention the intent distinctly, which could have led to subjective bias in our study.

## Conclusion

This review explores the complexity of PF and highlights the three leading unconscious motives: self-aggrandizing, seeking attention, and the assumption of a sick role. Exaggerations about health status, life events, education, and occupation present a challenge in rapport building, assessment, diagnosis, and management of a patient from a medical standpoint. Pseudologs assuming a different name or identity with dubious veracity and competency pose serious forensic ramifications and difficulties in standing trial under oath. Psychiatric comorbidities such as cluster B personality disorders, depressive disorders, trauma/stress disorders, somatic and factitious disorders, suicidality, and substance use (including alcoholism) are commonly associated with PF. Due to the strain that PF places on employment, societal relationships, and legal and medical systems, it is necessary to identify it early and educate physicians and law practitioners on how to deal with individuals affected by PF.

## Data Availability

Data are available upon reasonable request from the authors.

## Research registration unique identifying number (UIN)

This review was not registered, and a protocol was not prepared.

## Other relationships

All authors have declared that there are no other relationships or activities that could appear to have influenced the submitted work.

## References

[1] King BH Ford CV. Pseudologia fantastica. Acta Psychiatr Scand 1988;77:1–6. 3279719 10.1111/j.1600-0447.1988.tb05068.x

[2] Ford CV King BH Hollender MH. Lies and liars: psychiatric aspects of prevarication. Am J Psychiatry 1988;145:554–62. 3282449 10.1176/ajp.145.5.554

[3] Hardie TJ Reed A. Pseudologia fantastica, factitious disorder and impostership: a deception syndrome. Med Sci Law 1998;38:198–201. 9717367 10.1177/002580249803800303

[4] Frierson RL Joshi KG. Implications of pseudologia fantastica in criminal forensic evaluations: a review and case report. J Forensic Sci 2018;63:976–79. 28810073 10.1111/1556-4029.13616

[5] APA (American Psychiatric Association). Diagnostic and Statistical Manual of Mental Disorders (DSM-5). Booksmith Publishing LLC; 2021.

[6] Deutsch H Roazen P. On the pathological lie (Pseudologia Phantastica). J Am Acad Psychoanal 1982;10:369–86. 7107446 10.1521/jaap.1.1982.10.3.369

[7] Kainth T Gunturu S. Pseudologia Fantastica. In: StatPearls. StatPearls Publishing; 2025. Accessed 13 January 2025. http://www.ncbi.nlm.nih.gov/books/NBK606104/39163430

[8] DiSantostefano J. International Classification of Diseases 10th Revision (ICD-10). J Nurse Pract 2009;5:56–57.

[9] Bass C Jones D. Psychopathology of perpetrators of fabricated or induced illness in children: case series. Br J Psychiatry J Ment Sci 2011;199:113–18. 10.1192/bjp.bp.109.074088 21804147

[10] Hoyer TV. Pseudologia fantastica: a consideration of “the lie” and a case presentation. Psychiatr Q 1959;33:203–20. 14403524 10.1007/BF01575451

[11] Snyder S. Pseudologia fantastica in the borderline patient. Am J Psychiatry 1986;143:1287–89. 3766792 10.1176/ajp.143.10.1287

[12] Leung CM Lai K Shum K. Pseudologia fantastica and gender identity disturbance in a Chinese male. Aust N Z J Psychiatry 1995;29:321–23. 7487799 10.1080/00048679509075929

[13] Gogineni RR Newmark T. Pseudologia fantastica: a fascinating case report. Psychiatr Ann 2014;44:451–54.

[14] Wiersma D. On pathological lying. J Pers 1933;2:48–61.

[15] Teaford T Shaw RJ Reiss A. Pseudologia fantastica associated with pervasive developmental disorder. Psychiatry 2002;65:165–71. 12108140 10.1521/psyc.65.2.165.19932

[16] Korenis P Gonzalez L Kadriu B. Pseudologia fantastica: forensic and clinical treatment implications. Compr Psychiatry 2015;56:17–20. 25280799 10.1016/j.comppsych.2014.09.009

[17] Thom R Teslyar P Friedman R. Pseudologia fantastica in the emergency department: a case report and review of the literature. Case Rep Psychiatry 2017;2017:8961256. 28573061 10.1155/2017/8961256 PMC5442346

[18] Lidz T Miller JM. Muscular atrophy and pseudologia fantastica associated with islet cell adenoma of the pancreas. Arch Neurol Psychiatry 1949;62:304–13. 10.1001/archneurpsyc.1949.02310150051006 18147945

[19] Mitchell D Francis JP. A case of factitious disorder presenting as alcohol dependence. Subst Abuse 2003;24:187–89. 10.1080/08897070309511547 12913367

[20] Ford CV. The Munchausen syndrome: a report of four new cases and a review of psychodynamic considerations. Int J Psychiatry Med 1973;4:31–45. 4709440 10.2190/qe7c-d4jy-w3lq-y34c

[21] Dunn WS. Pseudologia phantastica, or pathological lying, in a case of hysteria with moral defect. J Ment Sci 1916;62:595–99.

[22] Birch CD Kelln BRC Aquino EPB. A review and case report of pseudologia fantastica. J Forensic Psychiatry Psychol 2006;17:299–320.

[23] Feldman MD. Factitious disorders in children and adolescents. Psychiatry Edgmont 2006;3:10–11. PMC2990619 21103173

[24] Grey JS Durns T Kious BM. Pseudologia fantastica: an elaborate tale of combat-related PTSD. J Psychiatr Pract 2020;26:241–45. 32421295 10.1097/PRA.0000000000000462

[25] Kerns LL. Falsifications in the psychiatric history: a differential diagnosis. Psychiatry 1986;49:13–17. 3704020 10.1080/00332747.1986.11024303

[26] Sale I Burvill J Kalucy R. Munchausen syndrome in a psychiatric setting: three case reports. Aust N Z J Psychiatry 1979;13:133–38. 291425 10.3109/00048677909159125

[27] Korkeila JA Martin TE Taiminen TJ. Clarification of pseudologia fantastica: a study of two cases of fantastic pseudology. Nord J Psychiatry 1995;49:367–71.

[28] Barocas D Difede J Viederman M. A case of chronic factitious disorder presenting as repeated, self-inflicted burns. Psychosomatics 1998;39:79–80. 9538680 10.1016/S0033-3182(98)71387-8

[29] Bauer M Boegner F. Neurological syndromes in factitious disorder. J Nerv Ment Dis 1996;184:281–88. 8627273 10.1097/00005053-199605000-00003

[30] Matas M Marriott A. The girl who cried wolf: pseudologia phantastica and sexual abuse. Can J Psychiatry Rev Can Psychiatr 1987;32:305–09. 10.1177/070674378703200412 3607712

[31] Dike CC. A radical reexamination of the association between pathological lying and factitious disorder. J Am Acad Psychiatry Law 2020;48:431–35. 33361173 10.29158/JAAPL.200074-20

[32] Weston WA Dalby JT. A case of pseudologia fantastica with antisocial personality disorder. Can J Psychiatry Rev Can Psychiatr 1991;36:612–14. 10.1177/070674379103600814 1742719

[33] Fink P Jensen J. Clinical characteristics of the munchausen syndrome. A review and 3 new case histories. Psychother Psychosom 1989;52:164–71. 2486395 10.1159/000288319

[34] Langer R. When the patient does not tell the truth. Psychoanal Soc Work 2010;17:1–16.

[35] Hekim A. An investigation at the point where mythomania meets manipulative lie: a forensic case. Dusunen Adam J Psychiatry Neurol Sci 2022. doi:10.14744/DAJPNS.2022.00176

[36] Green H James RA Gilbert JD. Medicolegal complications of pseudologia fantastica. Leg Med Tokyo Jpn 1999;1:254–56. 10.1016/s1344-6223(99)80046-7 12935477

[37] Newmark N Adityanjee N Kay J. Pseudologia fantastica and factitious disorder: review of the literature and a case report. Compr Psychiatry 1999;40:89–95. 10080254 10.1016/s0010-440x(99)90111-6

[38] Olisova OY Snarskaya ES Smirnova LM. Dermatitis artefacta: self-inflicted genital injury. Int Med Case Rep J 2019;12:71–73. 30936755 10.2147/IMCRJ.S192522 PMC6429999

[39] Parker PE. A case report of Munchausen syndrome with mixed psychological features. Psychosomatics 1993;34:360–64. 8351312 10.1016/S0033-3182(93)71871-X

[40] Sharrock R Cresswell M. Pseudologia Fantastica: a case study of a man charged with murder. Med Sci Law 1989;29:323–28. 2586275 10.1177/002580248902900412

[41] Moore K. Social work’s role with patients with munchausen syndrome. Soc Work 1995;40:823–25.

[42] Burt C. The analysis of temperament. Br J Med Psychol 1938;17:158–88.

[43] Burt C. The Young Delinquent. D. Appleton; 1925.

[44] Capraro V. Gender differences in lying in sender-receiver games: a meta-analysis. Judgm Decis Mak 2018;13:345–55.

[45] Friesen L Gangadharan L. Individual level evidence of dishonesty and the gender effect. Econ Lett 2012;117:624–26.

[46] Gerlach P Teodorescu K Hertwig R. The truth about lies: a meta-analysis on dishonest behavior. Psychol Bull 2019;145:1–44. 30596431 10.1037/bul0000174

[47] Elaad E Gonen-Gal Y. Face-to-face lying: gender and motivation to deceive. Front Psychol 2022;13:820923. 35391990 10.3389/fpsyg.2022.820923 PMC8982912

[48] Healy W Healy MT. Pathological lying, accusation, and swindling a study in forensic psychology. Gault R H Crossley F B Garner J W Eds Patterson Smith Repr Ser Criminol Law Enforc Soc Probl Montclair N J Patterson Smith 1969;Cases 1-12:22–27.

[49] Dupré E. Mythomanie infantile. Un cas de fugue, suivie de fabulation by Dupré, (Dr.).: très bon Couverture souple (1909) Edition originale | librairie Diona. 1909. Accessed 17 April 2024. https://www.abebooks.co.uk/first-edition/Mythomanie-infantile-cas-fugue-suivie-fabulation/31762055569/bd

[50] Altmann T Roth M. Vulnerable women and grandiose men? A 2D:4D study on the links between narcissism and prenatal estrogen/testosterone exposure in women and men. Personal Individ Differ 2024;229:112756.

[51] Green A MacLean R Charles K. Recollections of parenting styles in the development of narcissism: the role of gender. Personal Individ Differ 2020;167:110246.

[52] Hart B. Psychopathology, Its Development and Its Place in Medicine. The Cambridge University Press; 1939. https://assets.cambridge.org/97811076/93647/excerpt/9781107693647_excerpt.pdf

[53] Karpman B. Lying; a minor inquiry into the ethics of neurotic and psychopathic behavior. J Crim Law Criminol Am J Police Sci 1949;40:135–57. 18140421

[54] Bromberg W. The liar in delinquency and crime - Google Scholar. Nerv Child 1942;1:351–57.

[55] Turner MA. Factitious disorders: reformulating the DSM-IV criteria. Psychosomatics 2006;47:23–32. 16384804 10.1176/appi.psy.47.1.23

